# Heterogeneity of Hematological Response to Hypoxia and Short-Term or Medium-Term Bed Rest

**DOI:** 10.3389/fphys.2021.777611

**Published:** 2021-12-14

**Authors:** Joshua T. Royal, Ola Eiken, Michail E. Keramidas, Adam C. McDonnell, Igor B. Mekjavic

**Affiliations:** ^1^Environmental Physiology and Ergonomics Lab, Department of Automation, Biocybernetics and Robotics, Jozef Stefan Institute, Ljubljana, Slovenia; ^2^Jožef Stefan International Postgraduate School, Ljubljana, Slovenia; ^3^Division of Environmental Physiology, Swedish Aerospace Physiology Center, KTH Royal Institute of Technology, Solna, Sweden; ^4^Department of Biomedical Physiology and Kinesiology, Simon Fraser University, Burnaby, BC, Canada

**Keywords:** bed rest, hypoxia, hematology – red cells, variability – individual, inactivity

## Abstract

Hematological changes are commonly observed following prolonged exposure to hypoxia and bed rest. Typically, such responses have been reported as means and standard deviations, however, investigation into the responses of individuals is insufficient. Therefore, the present study retrospectively assessed individual variation in the hematological responses to severe inactivity (bed rest) and hypoxia. The data were derived from three-bed rest projects: two 10-d (LunHab project: 8 males; FemHab project: 12 females), and one 21-d (PlanHab project: 11 males). Each project comprised a normoxic bed rest (NBR; P_I_O_2_=133mmHg) and hypoxic bed rest (HBR; P_I_O_2_=91mmHg) intervention, where the subjects were confined in the Planica facility (Rateče, Slovenia). During the HBR intervention, subjects were exposed to normobaric hypoxia equivalent to an altitude of 4,000m. NBR and HBR interventions were conducted in a random order and separated by a washout period. Blood was drawn prior to (Pre), during, and post bed rest (R1, R2, R4) to analyze the individual variation in the responses of red blood cells (RBC), erythropoietin (EPO), and reticulocytes (Rct) to bed rest and hypoxia. No significant differences were found in the mean ∆(Pre-Post) values of EPO across projects (LunHab, FemHab, and PlanHab; *p*>0.05), however, female EPO responses to NBR (Range - 17.39, IQR – 12.97 mIU^.^ml^−1^) and HBR (Range – 49.00, IQR – 10.91 mIU^.^ml^−1^) were larger than males (LunHab NBR Range – 4.60, IQR – 2.03; HBR Range – 7.10, IQR – 2.78; PlanHab NBR Range – 7.23, IQR – 1.37; HBR Range – 9.72, IQR – 4.91 mIU^.^ml^−1^). Bed rest duration had no impact on the heterogeneity of EPO, Rct, and RBC responses (10-d v 21-d). The resultant hematological changes that occur during NBR and HBR are not proportional to the acute EPO response. The following cascade of hematological responses to NBR and HBR suggests that the source of variability in the present data is due to mechanisms related to hypoxia as opposed to inactivity alone. Studies investigating hematological changes should structure their study design to explore these mechanistic responses and elucidate the discord between the EPO response and hematological cascade to fully assess heterogeneity.

## Introduction

Due to the likelihood that future space vehicles and habitats, for logistical reasons, will be hypoxic (Bodkin et al., 2006; Norcross et al., 2013), our program of research (Keramidas et al., 2016b, 2017; McDonnell et al., 2019a; Mekjavic et al., 2020) investigated the effects of hypoxia on the known adaptation of physiological systems to short- and medium-term bed rest (i.e., ground-based experiments simulating the inactivity and unloading of the weight-bearing limbs).

The hematological changes that occur with severe inactivity were first reported by Taylor et al. (1945), who observed a 9.3% loss of blood volume, concomitant with a 15.5% contraction of plasma volume (PV) in healthy young males as a consequence of three-weeks of bed rest. Their results also revealed significant individual variability in the hematological responses to bed rest but were not explored. The observed normoxic bed rest (NBR)-induced hypovolemia was attributed to the prolonged cephalad fluid shift (CFS) that stimulates central volume carotid, aortic and cardiac receptors, releasing atrial natriuretic peptide (ANP) in turn causing diuresis and natriuresis and a resultant decrease in PV (Fortney et al., 2010). Renal release of the hormone erythropoietin (EPO) is inhibited by the resultant increase in central venous pressure during CFS bed rest (Kirsch et al., 1984; De Santo et al., 2005). Gunga et al. (1996) reported a rapid decline of EPO in the initial 24h (*p*<0.01) due to the initial increase in central venous pressure. Thereafter it returns gradually to pre-bed rest levels. Additionally, despite the EPO suppression found in the first 24h of NBR, some individuals experience concomitant increases in the concentrations of reticulocytes (Rct) and red blood cells (RBC; Ryan et al., 2016).

Reduced oxygen (O_2_) availability in the tissues resulting from a lower partial pressure of O_2_ in the ambient air stimulates the renal release of EPO (Scholz et al., 1990), which in turn promotes erythropoiesis in the bone marrow. When a red blood corpuscle has matured to a Rct, it is released into the circulating blood. The fraction of Rcts is typically 0.5–2.5% of the total RBCs circulating in the blood (Koepke and Koepke, 1986; Banfi et al., 2006). Once matured to RBC, the blood’s O_2_ carrying capacity is increased due to the rise in total RBC volume. PV decreases during hypoxic acclimation; however, studies that report this variable tend to report a large degree of interstudy variability (Sawka et al., 1996; Heinicke et al., 2003). Theoretically, as PV reduction is seen in both prolonged hypoxia and bed rest *via* different mechanisms, hypoxic bed rest (HBR) should produce compounded PV loss compared to hypoxia or bed rest alone (Loeppky et al., 1993; Keramidas et al., 2016a).

The concept of individuals being either responders or non-responders in response to an intervention is commonplace in physiology. Categorization of individuals into these groups is based on observing a response that exceeds the typical error of the measurement (Montero and Lundby, 2017). Ge et al. (2002), reported substantial individual variability in the EPO responses after several 24-h hypoxic exposures at a range of simulated altitudes. The authors also commented that individuals that had the largest responses to lower simulated elevations also had the largest responses to higher altitudes. Similar mean increases and a significant correlation between individual’s hemoglobin mass responses after normobaric and hypobaric hypoxic live high train low interventions have been reported (Hauser et al., 2017). A moderate altitude (~2100m) training camp attended by 12 Australian-Football players on 2 consecutive years found that individuals’ EPO responses to the same stimuli were not consistent from one year to the next (McLean et al., 2013). Of note, this finding was also present in the variability in the hemoglobin mass response of Finnish endurance athletes (Nummela et al., 2020), and German elite swimmers (Wachsmuth et al., 2013), both attributing this intraindividual variability to the lack of consistency and monitoring of athletes prior to each altitude exposure. Therefore, categorization of individuals into “responders” or “non-responders” after a single intervention is unreasoned and should not be considered an unchanging and distinguishable trait. Each of these studies’ authors stress the importance of individual evaluation of hematological variables in response to hypoxic exposures.

Levels of estrogen and progesterone change over the course of the menstrual cycle. Estrogen typically peaks during the late follicular phase, close to the start of ovulation, driving plasma volume expansion (Øian et al., 1987). However, in the late luteal phase, levels of progesterone increase, causing natriuresis and resulting in body fluid loss (Maffei et al., 1999). Reports investigating hematological changes during the menstrual cycle have described reductions (Vellar, 1974; Javaid et al., 2007; Ofojekwu et al., 2013), or no change (Kim et al., 1993; Lebrun et al., 1995) in Hb concentration during the early follicular compared to the luteal phase. However, it should be noted that Hb concentration’s stability is dependent upon the intra-extracellular fluid movements. Fortney et al. (1994), in a review of their previous series of studies, reported large fluctuations of PV and red cell mass within the menstrual cycle. The absolute PV and red cell mass were measured using a technetium radioisotope technique during the follicular and luteal phases of each woman’s menstrual cycle. A peak increase in PV was observed within two days of the estimated ovulation day, preceded by a decreased PV lasting 1 to 3days. Fortney et al. (1994) also reported that in both sexes, PV was significantly reduced post bed rest compared to pre; however, a greater degree of blood volume and PV loss was noted in males than females. While the menstrual cycle has a varying effect on PV, previous studies have reported no effect on red blood cell volume or hemoglobin mass (Chapman et al., 1997; Reeves et al., 2001; Malipatil and Patil, 2013; Aguree et al., 2020). Keller and colleagues (Keller et al., 2020) identified that although there was no significant change in hemoglobin mass across the menstrual cycle, the coefficient of variation (CoV) for hemoglobin mass over the duration of a single menstrual cycle was 4.1%, which is above the typical CoV commonly reported when using the carbon monoxide rebreathing technique (2.2%) and thus may be under-reported.

Common to the aforementioned studies is that individual variability is noted by presenting the standard deviation in the hematological changes observed in response to the bed rest, however, it is not discussed or expounded. Due to the potential for large individual variation in the responses to hypoxia and/or simulated microgravity, the recruitment of astronauts for future deep-space missions should consider individuals’ physiological systems’ responses to the adaptations to microgravity. Therefore, in conjunction with the European Astronaut Centre, a retrospective analysis was initiated on all hematological variables collected under the umbrella of the Slovene bed rest program conducted in the Planica facility. The present study aimed to assess the individual variability in the cascade of hematological responses to normoxic bed rest and HBR. We hypothesized that the process of acclimation as reflected in the hematological changes observed in subjects exposed to bed rest alone or in combination with hypoxia would not be the same for all participants.

## Methodology

### Ethical Approval

Subjects’ written informed consent was obtained prior to each project, and they were informed that they were free to withdraw their consent at any time. The procedures were approved by the Committee for Medical Ethics at the Ministry of Health (Republic of Slovenia; approval numbers: 205/2/11 and 88/04/12) conformed to the standards set by the Declaration of Helsinki (PlanHab: NCT02293772), except for the registration of the LunHab and FemHab projects in a database.

### Study Design

Data used for these analyses were derived from 3-bed rest projects: two 10-day bed rest projects (LunHab: male subjects; FemHab: female subjects) and one 21-day bed rest project (PlanHab: male subjects). Each project comprised two experimental interventions: normobaric normoxic bed rest (NBR) and normobaric HBR (simulated altitude of 4,000m, P_I_O_2_≈91mmHg). In each intervention, subjects were confined to one floor of the Olympic Sports Centre Planica (Rateče, Slovenia) situated at an altitude of 940m (partial pressure of inspired oxygen; P_I_O_2_=133mmHg). A horizontal position was maintained throughout all interventions, and subjects could only use one pillow for head support. Additionally, all activities were conducted in the horizontal position (i.e., hygiene, toilet, etc.), with the exception that during meals, they were allowed to rest on an elbow. Inclusion and exclusion criteria for PlanHab, LunHab, and FemHab have previously been described in detail (McDonnell et al., 2019a, 2020; Mekjavic et al., 2020) and followed the guidelines recommended by the European Space Agency (Heer et al., 2009). Concerning prior altitude exposure, subjects were excluded if they had been to altitudes above 2,000m within two months of the start of an intervention. The subjects’ physical characteristics are presented in Table 1. The detailed methodologies for each of these projects have been reported previously (Ciuha et al., 2015; Salvadego et al., 2016; Keramidas et al., 2016b; McDonnell et al., 2019a; Mekjavic et al., 2020).

**Table 1:** Physical characteristics of subjects that completed both NBR and HBR interventions in the 10-d LunHab, FemHab projects, and the 21-d PlanHab project.

Study	*n*	Sex	Age (yrs)	Height (*m*)	Weight (kg)
LunHab	8	M	23.4 (SD 1.7)	1.78 (SD 0.07)	74.1 (SD 14.1)
FemHab	12	F	26.1 (SD 3.7)	1.69 (SD 0.06)	59.5 (SD 8.8)
PlanHab	11	M	25.4 (SD 3.6)	1.80 (SD 0.04)	79.9 (SD 13.6)

The fraction of O_2_ in the Planica facility was maintained using a Vacuum-Pressure Swing Absorption system (VSPA, B-Cat, Tiel, The Netherlands). Samples of air from within each of the hypoxic rooms and common areas were analyzed at 15-min intervals for O_2_ and carbon dioxide content throughout the interventions. Should the O_2_ levels be above the target level, the introduction of a hypoxic gas mixture was initiated. In contrast, should the O_2_ levels decrease below the pre-set value, the system would terminate further delivery of hypoxic gas to that room. If the O_2_ did not return to the required level, delivery of external ambient (normoxic) air would be initiated, concomitant with the triggering of an audible alarm. In addition, each subject wore a personal portable (clip-on type) O_2_ analyzer (PGM-1100; Rae Systems, San Jose, California), providing immediate feedback of the F_I_O_2_ of the surrounding air and with an alarm alerting the user to the lower than anticipated O_2_ fraction. As a result of the VPSA monitoring system, the F_I_O_2_ was tightly controlled throughout all hypoxic interventions (LunHab: 0.144 SD 0.001, PlanHab: 0.141 SD 0 0.004, FemHab: 0.142 SD 0.001). As a result, the partial pressure of O_2_ in each project was the following: LunHab: 91.6 SD 0.14mmHg; PlanHab: 89.6 SD 0.4; FemHab: 90.4 SD 0.4mmHg).

### Measurements

Peripheral O_2_ saturation (SpO_2_) was measured daily in the morning after waking (07:00) in all interventions using a finger pulse oximeter (3,100 WristOx, Nonin Medicals, Minnesota, United States).

Venous blood was drawn from an antecubital vein at specific time points during each bed rest intervention; details of the exact blood sample timings may be found below in the Data Processing section. Blood samples were collected just after waking and prior to ambulation (relevant to the Pre and Post bed rest data collection) following an overnight fast. Approximately 200ml of blood was collected per participant in LunHab and FemHab, with 516.5ml of blood drawn per participant in PlanHab.

Blood samples for EPO analysis when collected (EDTA vacutainers) were allowed to coagulate for 20min, then centrifuged, and subsequently, aliquoted serum was frozen at −80°C for future analyses. EPO concentration was determined by sandwich enzyme-linked immunoassay (Quantikine IVD EPO ELISA; R&D Systems, Minneapolis, MN) using 100μl of serum. Optical density was quantified on a SPECTRAmaxTM PLUS384 microplate spectrophotometer (Molecular Devices Corporation, 1,311 Orleans Drive, Sunnyvale, California) set at 450nm and corrected at 600nm. The estimated CoV of the analysis was 2.2%.

Hb, Hct, RBCs, and Rct counts were analyzed with an automated laser-based hematology analyzer (Advia 120; Siemens, Munich, Germany) within 8h of blood sampling using clinical laboratory standards. All hematological variables were determined in duplicate by researchers blinded to the nature of the interventions.

Changes in PV were estimated from the Dill and Costill equation using Hct and Hb values (Dill and Costill, 1974). This approach was deemed appropriate for qualitative uses for this manuscript due to the concomitant changes in plasma renin concentration highlighted during a previous analysis of the PlanHab data (Keramidas et al., 2016b). Thus, any differences in PV between HBR and NBR in the three projects indicate qualitative variations in the response and do not permit us to draw firm conclusions regarding PV changes’ exact magnitude.

### Data Processing

The current study is an amalgamation of the results from three research projects designed to assess the separate and combined effects of hypoxia and bed rest on multiple physiological systems and the participants’ psychological status. Therefore, the data analysis was not included in the original design of the studies, namely, to assess individual variation and the chronological changes in the hematological variables. The three projects were similar in design and protocol. The experimental schedules have been reported previously for the LunHab (McDonnell et al., 2019b) and FemHab (McDonnell et al., 2020) projects. Due to minor changes in the experimental schedules the hematological sampling frequency is not consistent across the three projects.

Each of the three projects consisted of three interventions where subjects would experience one of the three conditions (NBR, HBR, and Hypoxic Ambulatory). Due either to subject dropouts, methodical error or human error, the Hypoxic Ambulatory data were too incomplete to compare with the NBR and HBR interventions. A minimum washout period of one month and three months was instituted between interventions for the 10-d (LunHab and FemHab) and 21-d (PlanHab) projects, respectively. Due to the sample sizes involved and the possibility of carryover at some physiological level between interventions despite the washout periods, the current data set was considered inappropriate for quantifying true individual response (SD_IR_; Williamson et al., 2017). As a result, the data presented in this study does not allow us to make conclusions as to the source of the variability; however, the current study highlights the importance of providing measures of individual variability when presenting results. As a result, the primary purpose of the current manuscript was to investigate the variability in the presented data and highlight the importance of providing measures of individual variability in the hemopoietic cascade of adaptation to bed rest.

The blood sampling draws for each project were:

- LunHab: Pre (Day-1) and Post (Day R1).
- FemHab: Pre (Day-2), During (Days 2 and 6), and Post (Days R1 and R2).
- PlanHab: Pre (Day-2), During (Days 2, 5, 14, and 21), and Post (Days R2 and R4).

On Day 1 of each intervention, subjects woke at 07:00, and continued with their assigned daily routine, following which they entered into the intervention, either HBR or NBR at 09:00. The subjects then conducted 10 (240h: LunHab & FemHab) or 21 (504h: PlanHab) days in that intervention. Thus, upon waking on the morning of R1, prior to reambulation at 09:00, the subjects were still in their designated intervention when a blood sample was collected at 07:00. Therefore, as there was no R1 data collection point for PlanHab, in order to ensure all Post blood draws were collected before reambulation, the Post values were collected on R1 for both LunHab and FemHab (R1) and Day 21 for PlanHab.

### Statistical Analyses

Data are expressed as individual responses, mean and SD, or as ranges and interquartile values. Statistical analyses were undertaken using SPSS (Version. 25, IBM, New York, United States) with significance set as *p*≤0.05. To assess whether significant statistical change had occurred in the pre- to post-intervention hematological values, a paired samples *t*-test was applied to the means. In the current analyses, subjects who completed both NBR and HBR interventions were included and paired-samples *t*-tests were used to distinguish differences between Δ(Pre-Post) values.

A One-way ANOVA was used to assess for significance between the NBR and HBR Δ(Pre-Post) values between projects (e.g., LunHab NBR vs. FemHab NBR vs. PlanHab NBR). The between variable relationship strength was calculated using Pearson’s or Spearman’s correlation analysis. Correlation analysis was used to assess potential relationships between SpO_2_ and EPO throughout the intervention. In all studies, potential relationships between pre-intervention hematological values and both absolute and relative degrees of change to post-intervention were investigated. Correlation coefficients were applied as recommended (Cohen, 2013; strong ≥0.60; moderate ≥0.40 −<0.59; weak ≥0.20 −<0.39).

A two-way repeated measures ANOVA was employed to assess the effect of time (Pre– vs. Post-bed rest) and condition (normoxia and hypoxia) within each bed rest project (LunHab, FemHab, and PlanHab). A two-way mixed-model ANOVA was employed to determine whether differences in the hematological markers existed due to the duration of comparable interventions (FemHab and PlanHab). In addition, post-hoc analyses using a Bonferroni corrected independent (between studies) and paired *t*-tests (within studies) were performed and reported where appropriate.

## Results

**Figure 1:**
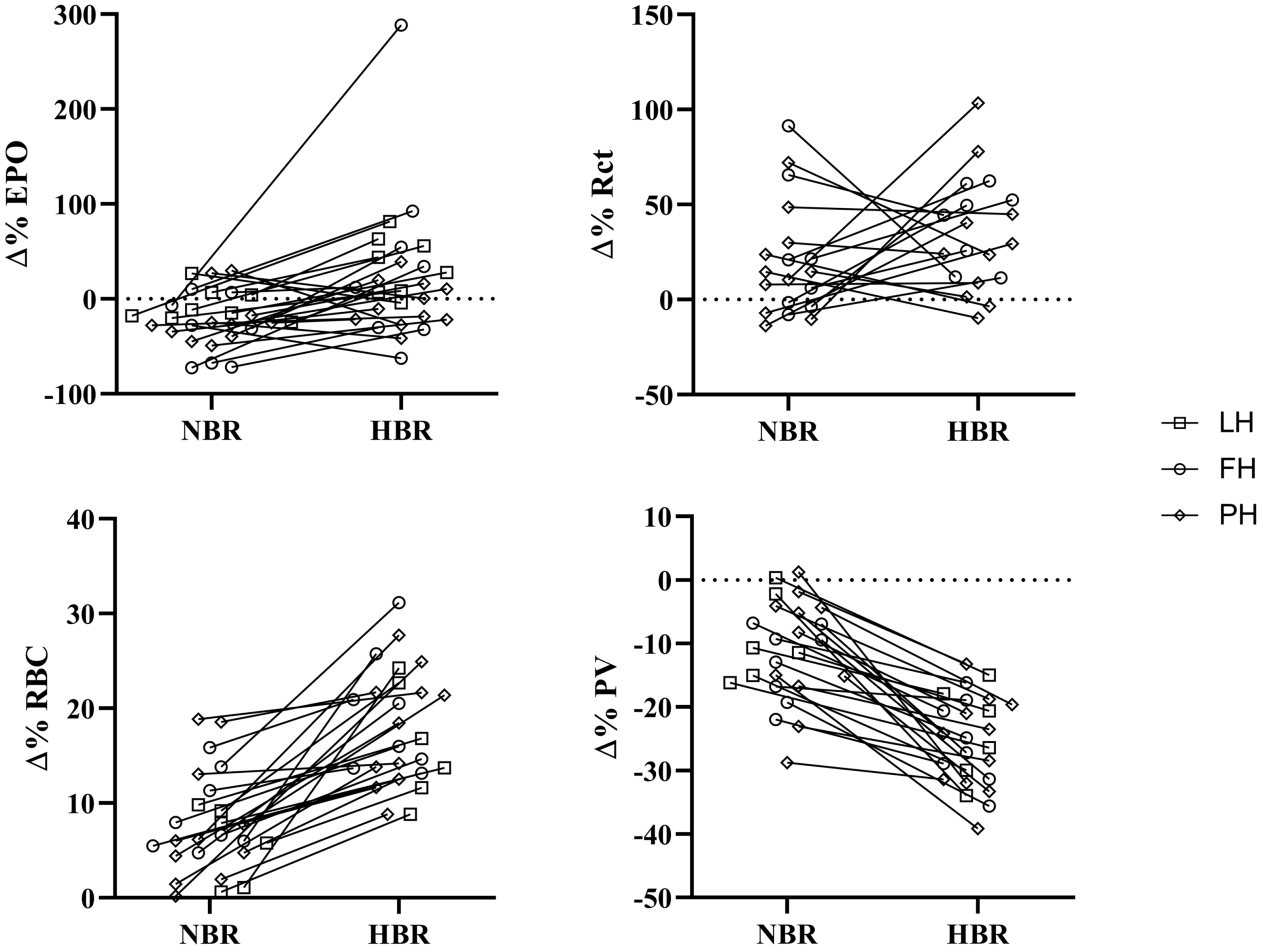
Individual relative (∆(Pre-Post)) responses of erythropoietin (∆%EPO), reticulocytes (∆%Rct), red blood cells (∆%RBC), and plasma volume (∆%PV) for NBR and HBR trials. Squares: LunHab; Circles: FemHab and Diamonds: PlanHab.

The Δ(Pre-Post) values [mean, (SD), overall range, and interquartile range] of EPO, Rct, RBC, and PV from each intervention for LunHab, PlanHab, and FemHab are presented in Table 2. The duration of bed rest did not have a statistical effect on any of the hematological variables, nor did subject sex (*p*>0.05). No correlations were found between the Δ(Pre-Post) bed rest hematological responses to NBR and HBR in EPO, Rct, or RBC (Figure 1).

**Table 2:** Bed rest Δ(Pre-Post) values in EPO concentration (ΔEPO), number of reticulocytes (ΔRct), number of red blood cells (ΔRBC), and plasma volume (ΔPV) during NBR and HBR interventions in all three projects.

		LunHab			FemHab			PlanHab		
		NBR	HBR	*p* ^†^	NBR	HBR	*p* ^†^	NBR	HBR	*p* ^†^
ΔEPO (mIU^.^ml^−1^)	Mean	−0.59^†^^,^^‡^	2.18^*^^,^^†^	0.036	−6.06^*^^,^^‡^	1.70	0.179	−2.21^*^^,^^†^	−1.28^†^	0.504
	SD	1.51	2.22		6.79	13.83		2.45	3.11	
	Range	4.60	7.10		17.39	49.00		7.23	9.72	
	IQR	2.03	2.78		12.97	10.91		1.37	4.91	
	p^*^	0.306	0.028		0.040	0.739		0.014	0.204	
ΔRct (×10^9^.L^−1^)	Mean	N/A			16.40	27.38^*^	0.364	8.94^*^	22.92^*^	0.138
	SD				25.53	13.62		12.37	23.30	
	Range				77.70	36.00		36.40	74.10	
	IQR				29.75	26.75		22.50	40.10	
	*p* ^*^				0.112	0.001		0.038	0.009	
ΔRBC (×10^12^.L^−1^)	Mean	0.28^*^^,^^†^	0.81^*^^,^^†^	0.016	0.40^*^^,^^†^	0.88^*^^,^^†^	0.002	0.38^*^^,^^†^	0.92^*^^,^^†^	0.001
	SD	0.20	0.33		0.16	0.28		0.32	0.33	
	Range	0.48	0.83		0.44	0.67		1.03	1.02	
	IQR	0.41	0.64		0.29	0.56		0.51	0.56	
	*p* ^*^	0.018	0.018		<0.001	<0.001		0.003	0.009	
ΔPV (%)	Mean	−5.08	−13.30		−8.01	−15.86		−6.18	−14.51	
	SD	3.73	4.22		3.85	4.42		5.27	4.34	
	Range	8.86	11.18		10.34	12.50		15.72	14.86	
	IQR	7.65	7.80		7.25	7.30		7.49	6.81	

*LunHab - 6 males (8 for EPO), FemHab - 8 females, PlanHab - 11 males; IQR – Interquartile Range*.

* *denotes significance between Δ(Pre-Post) values for condition (p≤0.05)*.

† *denotes significance in Δ(Pre-Post) values within study between normoxic and hypoxic gases (p≤0.05)*.

‡ *denotes significance in the Δ(Pre-Post) EPO response in NBR between LunHab and FemHab*.

### SpO_2_ and EPO Response Relationship

**Figure 2:**
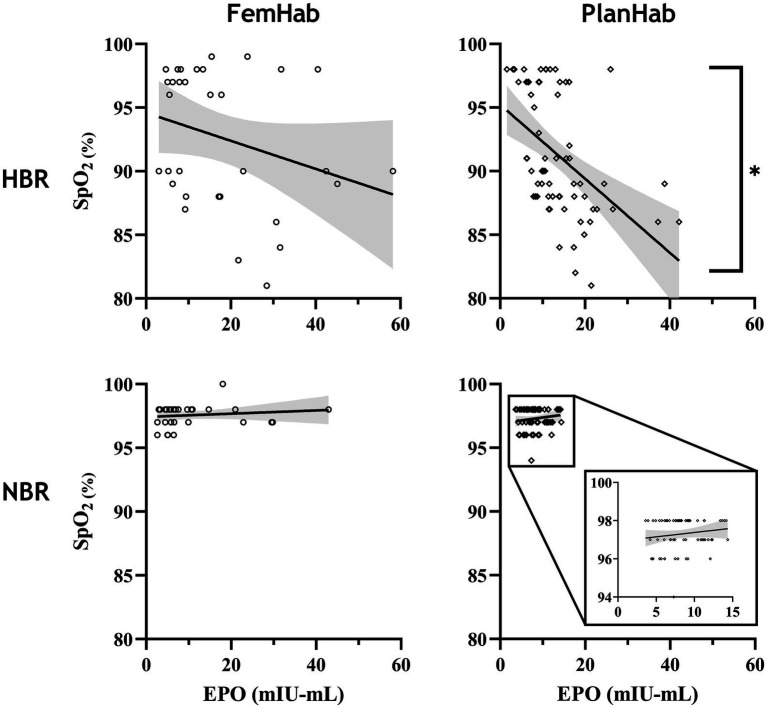
Correlation analysis between EPO and SpO2 values throughout the FemHab and PlanHab studies. SpO_2_ values were measured on the same days as EPO values were taken in each respective study. ^*^denotes significant correlation.

Correlation between SpO_2_ and EPO values throughout both the NBR and HBR interventions in FemHab and PlanHab were assessed (Figure 2). SpO_2_ presented in Figure 2 was collected on the same day as the corresponding blood draw. In PlanHab HBR, a significant moderate negative correlation was identified (*r*=− 0.561, *p*<0.001). No significant relationship was discovered in either FemHab intervention (HBR: *r*=− 0.252, *p*=0.117; NBR: *r*=0.200, *p*=0.271) or in PlanHab NBR (*r*=0.143, *p*=0.253).

### Changes in Erythropoietin

**Figure 3:**
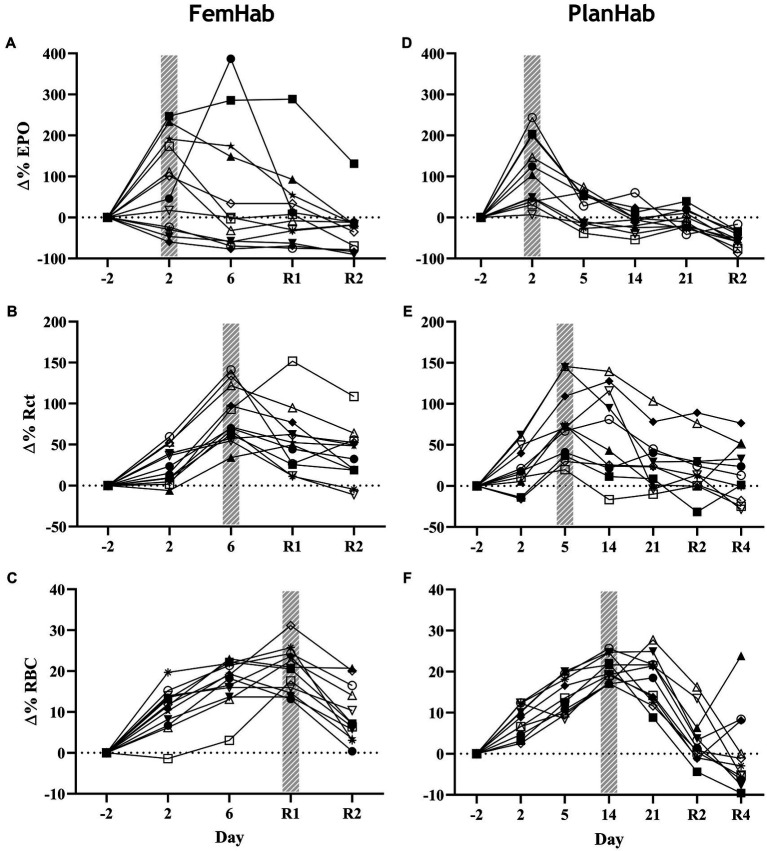
Baseline corrected relative individual changes (Δ%) of erythropoietin (∆EPO; **A,D)**, number of reticulocytes (∆Rct; **B,E)**, and number of RBCs (∆RBC; **C,F)** during the hypoxic bed rest (HBR) trials in the FemHab and PlanHab projects, respectively. The hatched columns indicate the highest mean group value for each variable. Each different symbol represents an individual subject that completed the intervention.

While mean EPO peaked in FemHab and PlanHab on Day 2 of HBR, in FemHab (Figure 3A), only six of the twelve subjects peaked on Day 2. Four of FemHab subjects’ peak EPO values did not increase above baseline for the HBR. All but one of FemHab’s subjects’ EPO values reduced to lower than baseline on Day R2.

The results of the PlanHab project (Figure 3D) indicate that eleven of the twelve subjects EPO peaked on Day 2. All subjects’ EPO reduced to lower than pre-HBR levels on Day R2. At the group level, significance was detected between Δ(Pre-Post) EPO values in LunHab HBR (*p*=0.028), FemHab NBR (*p*=0.040) and PlanHab NBR (*p*=0.014). The within-project ranges for EPO Δ(Pre-Post) during HBR were−0.5 to 6.6 mIU^.^ml^−1^ (LunHab), −25.34 to 23.66 mIU^.^ml^−1^ (FemHab) and−7.15 to 2.57 mIU^.^ml^−1^ (PlanHab). The within-project ranges for EPO Δ(Pre-Post) during NBR were−2.2 to 2.4 mIU^.^ml^−1^ (LunHab), −16.41 to 0.98 mIU^.^ml^−1^ (FemHab) and−4.47 to 1.77 mIU^.^ml^−1^ (PlanHab). Pearson’s correlation analyses showed that there were no correlations between NBR and HBR Δ(Pre-Post) EPO (LunHab: *r*=− 0.162, *p*=0.702; FemHab: *r*=0.469, *p*=0.241; PlanHab: *r*=−0.229, *p*=0.050).

### Changes in Reticulocytes

Group mean Rct peaked in FemHab on Day 6 and on Day 5 in PlanHab (Figures 3B,E) due to the sampling timeline. In FemHab, 2 of the subjects’ Rct values peaked on Day R2. However, in PlanHab, 4 subjects did not reach their maximum Rct concentration on the same day as the group mean peak value. After the Rct peak, these values gradually reduced to baseline levels in both projects.

At the intervention level, significance was discovered between pre and post Rct values in FemHab HBR (*p*=0.001), PlanHab NBR (*p*=0.038) and PlanHab HBR (*p*=0.009). From HBR Δ(Pre-Post), the range for each data set were 6.70 to 42.90×10^9.^L^−1^ (FemHab) and−8.2 to 65.9×10^9.^L^−1^ (PlanHab). No significance was found in the FemHab NBR intervention (*p*=0.112).

The inter-subject range for the changes in Rct NBR Δ(Pre-Post) -6.50 to 71.20×10^9.^L^−1^ (FemHab) and−8.70 to 27.70×10^9.^L^−1^ (PlanHab). Pearson’s correlation analyses showed there were no correlations between the Δ(Pre-Post) values in Rct for NBR and HBR (FemHab: *r*=− 0.280, *p*=0.501; PlanHab: *r*=− 0.218, *p*=0.520).

### Changes in Red Blood Cell Volume

RBC peaked in FemHab HBR on Day R1 despite half of the subjects’ individual peak scores occurring on Day 6 (Figure 3C). In PlanHab HBR, the peak in RBC was on Day 14 of the intervention, although 5 of the 11 subjects’ individual peaks were on a day other than Day 14 (Figure 3F). In both studies, after the group peak in RBC, values dropped to around that observed at baseline. At the group level, significance was discovered between Pre- vs. Post-bedrest RBC values in all data sets (LunHab, FemHab, and PlanHab) and conditions (NBR and HBR; *p*<0.05). NBR 0.03 to 0.51×10^12.^L^−1^ (LunHab), 0.24 to 68×10^12.^L^−1^ (FemHab) and 0.01 to 1.04×10^12.^L^−1^ (PlanHab). In HBR the Δ(Pre-Post) ranges for each data set were−0.11 to 1.27×10^12.^L^−1^ (LunHab), 0.62 to 1.29×10^12.^L^−1^ (FemHab) and 0.48 to 1.33×10^12.^L^−1^ (PlanHab). Pearson’s correlation analyses showed there were no correlations between the Δ(Pre-Post) changes in RBC for NBR and HBR (LunHab: *r*=0.161, *p*=0.761; FemHab: *r*=0.355, *p*=0.388; PlanHab: *r*=0.247, *p*=0.464).

### Baseline Corrected Responses

**Figure 4:**
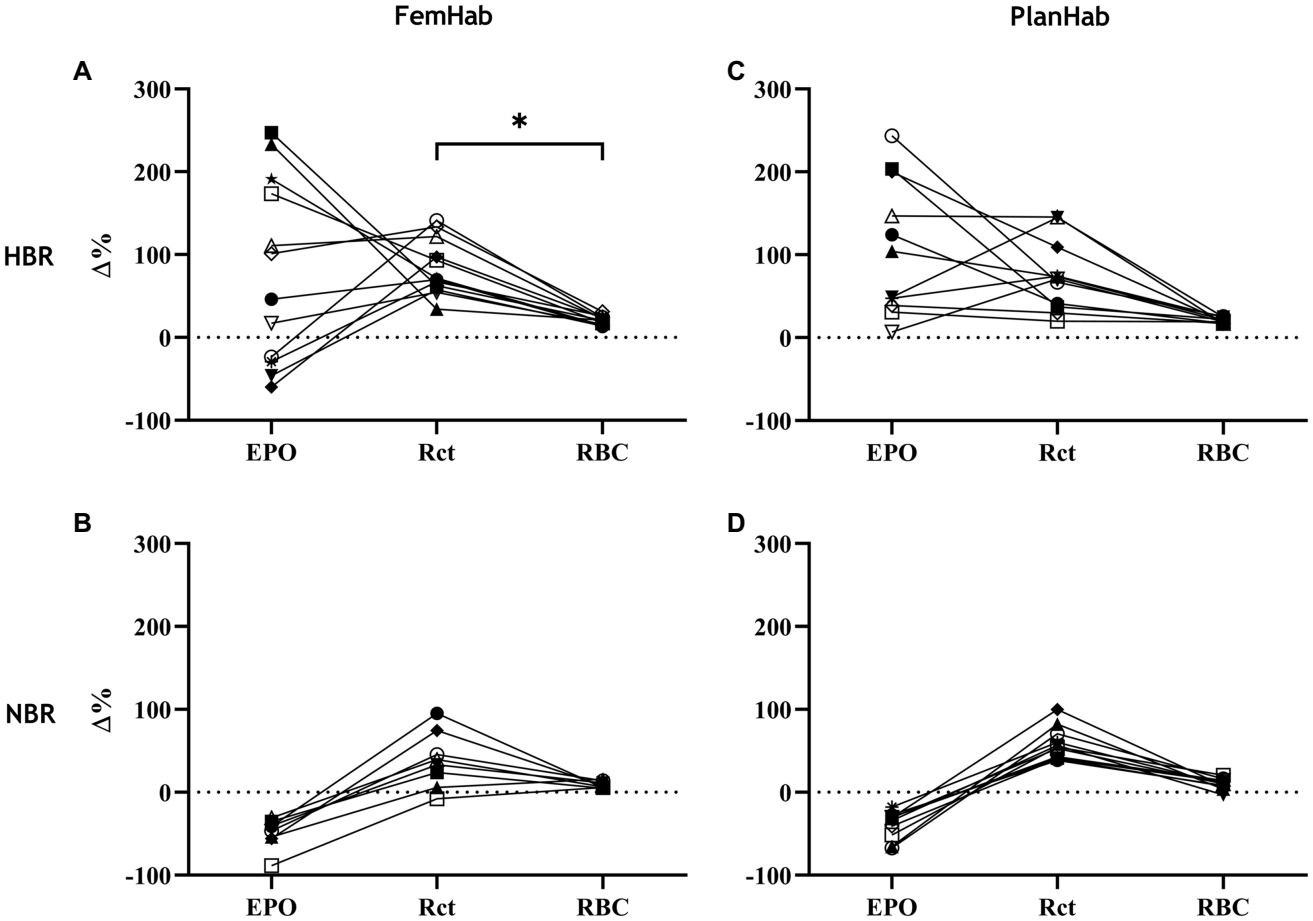
represents the relationships between individuals’ ∆EPO, ∆Rct, and ∆RBC in HBR **(A,C)** and NBR **(B,D)** in FemHab and PlanHab on the days of the highest mean group change (Δ%) for each variable during the HBR confinement. ^*^ denotes significant correlation between relative responses in FemHab HBR Δ(Pre-Post) Rct and RBC (*p*≤0.05). Each different symbol represents an individual subject that completed the intervention.

The only correlation between mean peaks in the measured hematological variables during Pearson’s correlation analysis was a significant positive moderate correlation between FemHab HBR Rct and RBC (*r*=0.597, *p*=0.040). No other correlation existed between the mean peaks in the measured hematological variables as either absolute or relative changes from baseline (*p*>0.05; Figure 4).

EPO, Rct and RBC significantly changed over the duration of the HBR in both FemHab and PlanHab (FemHab EPO: *F* (2.106, 23.166)=3.037, *p*=0.027, *η*^2^=0.216; FemHab Rct: *F* (2.007, 22.074)=18.823, *p*<0.001, *η*^2^=0.631; FemHab RBC: *F* (4, 44)=37.919, p<0.001, *η*^2^=0.775; PlanHab EPO: *F* (2.007, 20.068)=30.176, p<0.001, *η*^2^=0.751; PlanHab Rct: *F* (6, 60)=13.802, *p*<0.001, *η*^2^=0.580; PlanHab RBC: *F* (2.485, 24.850)=30.243, *p*<0.001, *η*^2^=0.752; Figure 3).

### Changes in Plasma Volume

**Figure 5:**
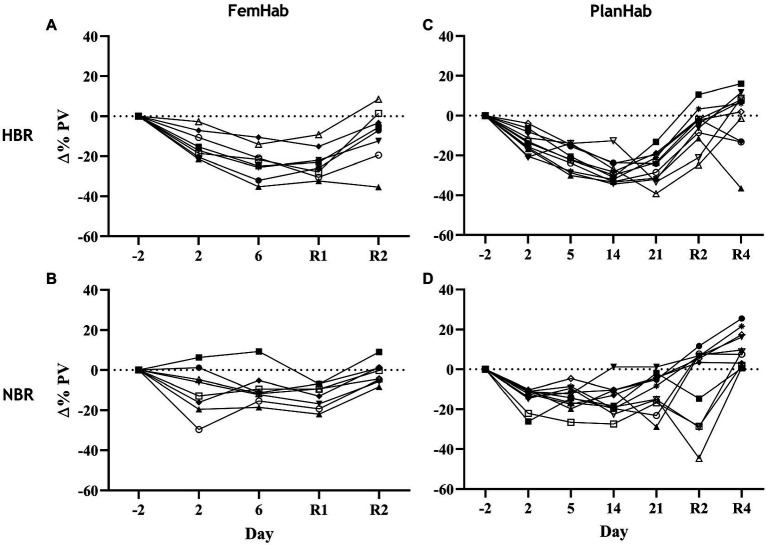
Individual changes in PV relative to pre-intervention baseline values during NBR and HBR interventions in FemHab **(A,B)** and PlanHab **(C,D)** studies. Each different symbol represents an individual subject that completed the intervention.

Any differences found in ΔPV between HBR and NBR in the three projects (LunHab, PlanHab, FemHab: Table 2; Figure 5) are purely speculative due to the calculation methods employed and do not permit us to draw firm conclusions regarding the exact magnitude of PV changes. Pearson’s correlation analyses revealed no correlations in the ΔPV between NBR and HBR (LunHab: *r*=0.209, *p*=0.691; FemHab: *r*=0.385, *p*=0.346; PlanHab: *r*=0.489, *p*=0.127).

## Discussion

It is well documented that both genotypic and phenotypic factors influence the responses of individuals exposed to hypoxia (Moore, 2001; Beall, 2014). The principal finding of the present study is that for the same hypoxic stimulus, these factors affect the cascade of hematological responses among inactive subjects differentially. Namely, for a given hypoxic exposure (approximately P_I_O_2_=90mmHg) of 10- and 21-day duration, we observed the resultant responses of arterial O_2_ saturation, which placed in motion the cascade of events from the release of EPO to the resultant increased production of Rcts and finally RBCs. The latter being essential for the ability of blood to bind O_2_. Hypoxic acclimatization and training protocols strive to achieve an optimal outcome of the last event in this cascade, namely an increase in RBCs. This is considered essential for maintaining the performance of lowlanders at high altitudes (i.e., alpinists), or for improving their sea-level performance (i.e., athletes). Surprisingly, the substantial individual variation observed in the first steps (SpO_2_, EPO) of the cascade in HBR, gradually diminishes towards the last step of the cascade (RBC), as evident in Figure 4.

### SpO_2_ and EPO Response Relationship

A significant negative correlation between SpO_2_ and EPO only existed in the PlanHab HBR intervention (Figure 2). FemHab NBR had a larger variation in EPO response than values at the same SpO2 in PlanHab, with some females eliciting a response equal or greater to that of males and females in HBR. In females, the hypoxic ventilatory response has been stated to be significantly more extensive during the luteal versus the follicular phase (Takano, 1984). As the hypoxic ventilatory response is an indicator of the chemosensitivity to hypoxia, this change in chemosensitivity may affect the EPO response at a given SpO_2_. Since no control was in place, and monitoring of females’ menstrual phase in the FemHab study was limited, it cannot be verified whether the females with exaggerated EPO responses during NBR, despite no change in SpO_2_, were also in the luteal phase at that time. The higher degree of variation in females’ EPO response may also be a contributing factor as to why no statistical correlation was observed in HBR during FemHab.

### Magnitude of the Hematological Responses

The EPO response to NBR and HBR in females appears to be considerably larger than in males (Table 2). Additionally, bed rest duration appears to have no impact on the heterogeneity of hematological responses (Table 2). Our analyses also show that the resultant hematological changes (Rct and RBC) that occur during NBR and HBR are not proportional to the EPO level when individual responses are considered. Considering hypoxia as the stimulus for the hematological changes, we demonstrate the heterogeneity of the cascade of responses to this stimulus, from arterial O_2_ saturation (Figure 2) to increased EPO release and production of Rct and RBC (Figure 3). The increase in EPO concentration (Figures 3A,D) within the first days of exposure is followed by an increase in Rct concentration (Figures 3B,E) by Days 5 (FemHab) and 6 (PlanHab), finally resulting in an increase in RBC (Figures 3C,F) by Days R1 (FemHab) and 14 (PlanHab). Qualitative analysis of the individual responses indicates a large degree of individual variation in these responses’ magnitude and kinetics. Finally, these responses need to be considered from the perspective of the plasma volume changes, which is largely affected by individual variation itself. Intriguingly, despite the large range of EPO responses observed in both studies, the range of RBC concentration, the last step in this cascade, is substantially lower. These issues are discussed in further detail below.

The magnitude of an individual’s relative EPO or Rct response is not necessarily indicative of the size of their relative Rct or RBC response, respectively (Figure 4). The reason the relative increases across the hematological variables are not consistent, could be due to scale relativity; however, the increases in these hematological variables are most likely primarily attributable to increases in hemoconcentration from hypovolemia. Rct fraction of total RBC volume is typically 0.5–2.5% (Koepke and Koepke, 1986; Banfi et al., 2006); therefore, an increase of 100% in Rct concentration would hypothetically only account for a consequential RBC increase of 1–5%. Reductions in RBC volume seen in Days R2 (FemHab), 21, and R2 (PlanHab) initially seem implausible due to the typical lifespan of circulating RBC and are most likely due to the sudden rise in PV seen in the latter half of the interventions (Figure 5).

The magnitude of EPO production is largely dependent on the level of an individual’s hypoxic stress (Chapman et al., 2014b); however, the increase in EPO level during a fixed hypoxic stimulus between subjects varies considerably (Ploszczyca et al., 2018), as demonstrated in Figures 2, 4. The differences in the spread of individual responses to NBR and HBR shown in Figure 4 indicate that the majority of variability seen in the hematological variables is due to the mechanisms responding to hypoxic acclimation rather than bed rest. Inter-individual variation in EPO response has previously been observed by Klausen et al. (1996), with some subjects having a serum EPO response almost 10x greater than others after 42-h at an altitude of 4,350m. Chapman et al., (2010) also found large variation in the EPO response when taking 26 elite distance runners to 2,500m elevation for 20h (ΔEPO – 2.9ng^.^ml^−1^ to 20.5ng^.^ml^−1^; −19.9 to 415.4%). Variation amongst subjects in the magnitude of the EPO response to a fixed hypoxic stimulus could potentially be influenced by a multitude of factors. Disparities in subjects’ carotid body chemosensitivity, hypoxic ventilatory drive, hemoconcentration, or renal blood flow at the moment of renal EPO release, and factors that are potentially hereditary traits, may explain why inter-individual variation is often more common than intra-individual variation (Collins et al., 1978; Scoggin et al., 1978).

### Hematological Kinetic Response

The timing of the mean group peaks in EPO, Rct, and RBC in Figure 3 concur with previous reports (Scholz et al., 1990; Banfi et al., 2006). A reduction in EPO level after the initial peak is apparent in both FemHab and PlanHab studies (Figures 3A,D), a finding that is in line with other altitude studies (Ploszczyca et al., 2018). However, the underlying mechanisms for this reduction are still not entirely clear. Lundby et al. (2009) noted that hypoxic inducible factor-1 (HIF-1) peaks within the first hours of hypoxic exposure and then reduces gradually to pre-hypoxic exposure levels. The authors speculated that the response to the initial hypoxic stimulus might diminish the degree of cellular hypoxia. Ploszczyca et al. (2018) have also suggested that the gradual reduction in EPO over prolonged hypoxic exposure is likely associated with the reduction in HIF-1. However, the rate of decrease in EPO is not equivalent to the lesser reduction in circulating Rcts (Rusko et al., 2004). Therefore, the authors suggested that the reduction in serum EPO after the initial peak is due to the establishment of an equilibrium between EPO production and consumption for Rct creation.

### Sex

Absolute and relative EPO responses had considerably greater inter-individual variability (Table 2; Figures 3, 4) in the female bed rest study than the two male bed rest studies, and no statistical mean differences in the relative responses. Distribution values (SD, range and IQR; Table 2) in the FemHab study are all larger in both NBR and HBR interventions compared to their male counterparts. The relative EPO responses of females to hypoxia in comparison to LunHab and PlanHab studies appear to have large variability, which diminishes when compared to previous studies with prolonged hypoxic stimuli (Ploszczyca et al., 2018). Females tend to have lower [Hb] than males, meaning their concentration of arterial O_2_ at any given O_2_ saturation is usually lower (Murphy et al., 2010). Chapman et al. (2010) speculated that this hypothetically means females have a larger EPO response to hypoxia than males. Although in the present study no differences were found in EPO between males and females at the group level, the variability in EPO response found in FemHab could be due to individuals’ being at different phases of their menstrual cycle as no control was implemented on menstrual cycle phase during the bed rest interventions. Stachenfeld (2008) suggested that estrogens play an important role in stabilizing body fluid volume. During a normal menstrual cycle, estrogens potentially function to counter the fluid loss effects of progestins, maintaining PV. In response to long-duration stress, such as a prolonged bed rest (greater than 5–7days) or weightlessness, estrogens are speculated to stabilize the vascular compartment (Fortney et al., 1988). A manner to potentially reduce EPO variability in females would be through control of estradiol and progesterone. Contraception has been shown to control levels of estrogen but not progesterone so is not ideal. The use of a gonadotropin releasing hormone agonist or antagonist for reproductive function suppression with a controlled administration or estradiol and progesterone would negate hormonal fluctuations (Stachenfeld, 2008), and in turn potentially reduce variability. Tracking the menstrual cycle and hormonal changes throughout the intervention would not change the level of variability however it would allow researchers to identify if larger EPO responses were concurrent with changes in either estradiol or progesterone.

Until the age of puberty, males and females have similar increases in Hbmass during their development. After puberty has begun, Hbmass increases exponentially in males, whilst remaining at a rate similar to pre-puberty in females (Prommer et al., 2018). It is believed the source of this change of rate is due to the role of androgens in males’ puberty (Hero et al., 2005). The introduction of testosterone causes a rightward shift in the EPO-Hb relationship curve as well as a new physiological “set point” (Bachman et al., 2014). Mancera-Soto et al. (2021), claims that during the most sensible phase of puberty, an increase in testosterone plasma of 1ng/ml is correlated to an increase in hemoglobin mass of ~65g. The effects of testosterone on individual variability in men compared to women; however, is currently unknown and would require further examination. Furthermore, Goodrich et al. (2020) demonstrated that variations in hemoglobin mass across groups of varying athleticism and sex were more closely related to lean body mass than whole body mass. Once normalized for lean mass, seen previously variations were greatly diminished. In addition, they also pointed to deficiency as a strong determinator of lower hemoglobin mass. Further, iron deficiency anemia was identified to be in far higher prevalence in women of childbearing age due to blood and iron loss during the menstrual cycle (Fernandez-Jimenez et al., 2020). Both of these mechanisms may explain to a certain extent the potential sex difference noted in the current manuscript.

### Plasma Volume Changes

Prolonged bed rest and hypoxia independently both cause reductions in PV (Table 2; Figure 5). Bed rest duration or subject sex do not appear to contribute to the inter-individual variability of the PV response. Bed rest’s horizontal positioning stimulates receptors in the upper body after the CFS, which, in turn, results in the release of ANP, causing diuresis and natriuresis. PV changes during hypoxic acclimation have considerable variability and appear to be mediated primarily by changes in oncotic pressure (Siebenmann et al., 2017b). The majority of the variability in the PV response to hypoxic acclimation is attributed to the variability in the concomitant changes in total circulating protein (TCP) during hypoxic acclimation. Young et al. (2019) speculated that this decrease in TCP could be attributed to both reduced plasma protein synthesis and leaking of plasma proteins into the extravascular space. The combination of both of these mechanisms results in approximately twice the reduction of PV in comparison to NBR alone at the intervention level (Table 2). The changes in PV seen in NBR have been speculated to modulate the production and release of EPO (Gunga et al., 1996). Keramidas et al. (Keramidas et al., 2016b) concluded that tissue O_2_ saturation is a primary mediator for the degree of renal EPO synthesis and that PV is a secondary factor. Bed rest duration appears to have little to no impact upon the amount of individual variation in PV seen in the Δ(Pre-Post) and day-to-day values (Table 2).

### Limitations

Using hemoglobin mass (Hb_mass_) as opposed to [Hb] concentration and RBC would eliminate the effects of PV retraction on measuring the variability in changes to blood O_2_ carrying capacity. Hemoglobin mass typically has a measurement error of around 2% in well trained research teams (Gough et al., 2011; Steiner and Wehrlin, 2011; Siebenmann et al., 2017a). The Dill and Costill (1974) equation, utilized in this study, is deemed appropriate for calculation of plasma and serum biomarkers; however, not for whole blood biomarkers (Matomäki et al., 2018). Future research should consider using a direct tracer-dilution method to study PV changes and draw firm conclusions as to the exact extent of PV changes.

The debate between hypo- and normobaric hypoxic continues and is sometimes overlooked when discussing hypoxic exposure in general. There are many discrepancies in the responses of physiological systems between normobaric hypoxia and hypobaric hypoxia which have been attributed to the differences in barometric pressure (Millet and Debevec, 2020) and may indeed be worth further study. Despite this, Hauser et al. (2016) found similar Hb_mass_ responses to live high train low interventions in normobaric and hypobaric hypoxia. Wide variability existed in individual responses to both intervention types after the same hypoxic dose and after 18days post-intervention. The heterogeneity of hematological responses to inactivity and hypobaric hypoxia may differ either in source or in magnitude to that of inactivity and normobaric hypoxia and require further investigation.

Another stipulation worth mentioning is that of the total 562 astronauts that have ventured to space between 1961 and 2020, the average age of astronauts during their first mission is 39.8years SD 5.28. It should also be noted that astronauts from the 2000s and 2010s are significantly older during their first missions than those before the turn of the century (Smith et al., 2020). The average ages of the participant groups used in this study were in their mid-twenties, and therefore may not be seen as a complete representation of aging astronauts, especially those completing their second and third missions. The effects of aging on serum EPO are known, with increased values with age potentially as a compensation mechanism for increased hemolysis or a rise in EPO resistance (Ershler et al., 2005). Whether these changes would affect the heterogeneity of hematological responses, however, is unknown.

## Conclusion

A group of individuals’ responses to a stimulus or intervention are typically reported as means and standard deviations. The current spike of interest in individual variation noted in recent years demonstrates that this analytical approach may mask the true range of individual responses potential led researchers to draw inappropriate inferences. Acknowledgment of this variability is essential to optimize personal future medical and physiological interventions. The recent pandemic of the severe acute respiratory syndrome coronavirus 2 (SARS-CoV-2) has highlighted the substantial heterogeneity observed in the development of systemic hypoxia among infected patients. The degree of hypoxia developed during the illness not only dictates whether hospitalization is required but also whether the induction of coma with supplemental O_2_ breathing is required for survival. Clearly, there is great individual variability in the ability of the body to respond and adapt to the severe hypoxia, which in extreme situations, is essential for survival. The current investigation of the hematological responses of inactive males and females exposed to the same magnitude and duration of a hypoxic stimulus demonstrated the substantial heterogeneity in the cascade of responses from arterial O_2_ saturation to RBC production. The individual variability in the EPO response to NBR and HBR in females appears to be considerably larger than in males, and the duration of bed rest appears to have no impact on the heterogeneity of the hematological responses. Our findings suggest that relative EPO responses are not sufficient indicators of the resultant increased production of Rcts and RBCs. The data would suggest that the majority of the variability seen in HBR is due to mechanisms responding to hypoxia rather than bed rest and inactivity. The significance of the current data is the identification and acknowledgment of large individual variability within the mechanistic response to hypoxia, thus creating justification to further investigate the sources and moderating factors of such variability.

## Data Availability Statement

The original contributions presented in the study are included in the article/supplementary material, further inquiries can be directed to the corresponding author.

## Ethics Statement

The studies involving human participants were reviewed and approved by Komisija za Medicinsko Etiko. The patients/participants provided their written informed consent to participate in this study.

## Author Contributions

MK, OE, AM, and IM designed and coordinated the original research. AM, MK, OE, and IM collected the data. JR analyzed data. JR drafted the manuscript. All authors critically read, edited, and approved the final manuscript.

## Funding

Financial support by the European Space Agency, Program for European Cooperating States (Contract No. 4000124642/18/NL/PG/gm: “Individual variation in human responses to prolonged bed red in Slovene bed rest program” and Contract No. 40001043721/11/NL/KML: “Planetary Habitat Simulation”), by the European Union Program FP7 (PlanHab project: 284438), by the Slovene Research Agency (Contract No. L3-3654), the Swedish National Space Agency (Contract No. 109/11:2) and internal funds from the Royal Institute of Technology (KTH).

## Conflict of Interest

The authors declare that the research was conducted in the absence of any commercial or financial relationships that could be construed as a potential conflict of interest.

## Publisher’s Note

All claims expressed in this article are solely those of the authors and do not necessarily represent those of their affiliated organizations, or those of the publisher, the editors and the reviewers. Any product that may be evaluated in this article, or claim that may be made by its manufacturer, is not guaranteed or endorsed by the publisher.
